# Design and Verification of a Digital Controller for a 2-Piece Hemispherical Resonator Gyroscope

**DOI:** 10.3390/s16040555

**Published:** 2016-04-20

**Authors:** Jungshin Lee, Sung Wook Yun, Jaewook Rhim

**Affiliations:** Inertial Sensors & Instruments, Agency for Defense Development, Bugyuseong daero 488 beon gi, Yuseong-Gu, Daejeon 305-152, Korea; swyun@add.re.kr (S.W.Y.); jylim@add.re.kr (J.R.)

**Keywords:** hemispherical resonator gyroscope (HRG), electromechanical gain, Duhamel integral, mode shape, multi-flexing, demodulation, modulation, force-to-rebalance (FTR) mode, pendulum variables, direct digital synthesizer (DDS)

## Abstract

A Hemispherical Resonator Gyro (HRG) is the Coriolis Vibratory Gyro (CVG) that measures rotation angle or angular velocity using Coriolis force acting the vibrating mass. A HRG can be used as a rate gyro or integrating gyro without structural modification by simply changing the control scheme. In this paper, differential control algorithms are designed for a 2-piece HRG. To design a precision controller, the electromechanical modelling and signal processing must be pre-performed accurately. Therefore, the equations of motion for the HRG resonator with switched harmonic excitations are derived with the Duhamel Integral method. Electromechanical modeling of the resonator, electric module and charge amplifier is performed by considering the mode shape of a thin hemispherical shell. Further, signal processing and control algorithms are designed. The multi-flexing scheme of sensing, driving cycles and x, y-axis switching cycles is appropriate for high precision and low maneuverability systems. The differential control scheme is easily capable of rejecting the common mode errors of x, y-axis signals and changing the rate integrating mode on basis of these studies. In the rate gyro mode the controller is composed of Phase-Locked Loop (PLL), amplitude, quadrature and rate control loop. All controllers are designed on basis of a digital PI controller. The signal processing and control algorithms are verified through Matlab/Simulink simulations. Finally, a FPGA and DSP board with these algorithms is verified through experiments.

## 1. Introduction

A Hemispherical Resonance Gyroscope (HRG) is a Coriolis Vibrating Gyroscope (CVG), which measures the angle or angular velocity using the Coriolis force generated by the rotational motion [1]. HRGs are suitable for miniaturization and high precision due to their simple structure made up of five components and easy production process. Particularly, since it is a sensor made of quartz material, which has excellent material properties, using the solid state wave phenomenon, it guarantees high reliability and a long-term lifespan. In addition, it has the advantage that it can be used in the angular velocity mode or the integral angular velocity mode according to the control technique and the electronic circuit used, without requiring any structural changes to the sensor [1].

The development of HRG technology, which started in the 1970s, has been conducted actively in the advanced countries such as the United States (Northrop Grumman), France (SAGEM), Russia (RDC, Medicon), *etc.* for the purpose of its use in the attitude controllers of satellites and spaceships, long-term navigation systems for strategic missiles, submarines, *etc.*, where long-term reliability is important [2].

The United States (Northrop Grumman) is currently producing miniature HRGs, the size of golf balls, based on the existing HRG 130P from 2012. This is the results of design improvement that converts the 3-piece system, in which the forcer and pickoff are divided, into a 2-piece system, in which the outer forcer is removed, and the process stage is reduced using the multi-flexing method [3]. The multi-flexing method involves obtaining the sensing and driving signal of the x-axis and y-axis using the same element by switching within the specified cycle and is suitable for high precision and low maneuverability systems like satellite systems. In addition, the SIGMA 20 navigation system, according to data made public by France in 2013, is under development based on the 2-piece HRG, which applies the multi-flexing technique to a resonator of 20 mm diameter, showing that it can be utilized in ground navigation systems used in rough environments focusing on the characteristics of the HRG, which can endure thermal and mechanical stress [4].

In the meantime, the traditional control method controls the x-axis to be major axis of the elliptical trajectory expressing the pendulum variables from the axis where the excitation and sensing electrodes are arranged. On the contrary, the differential control method controls the axis which forms a 45° angle with the x-axis, to be the major axis of the elliptical trajectory, This method has the advantage that it can remove the common mode error for both the x-axis and the y-axis and is easy to switch to the integral angular velocity mode [5].

As such, next generation HRGs will evolve into representative gyros, which can materialize the demands for subminiature structure, high precision and high reliability by the development of 2-piece systems applying multi-flexing methods, differential control algorithms, *etc.* To develop such subminiature and high precision HRGs, advanced core processes such as the production of low-loss hemispherical resonators and electrode blocks, low-stress heterojunctions, the balancing and tuning, high degrees of vacuum packaging, *etc.* and electronic module technologies such as low-noise pre-amplifiers, FPGA-based high precision digital signal processing and control circuits, error modelling and compensation techniques, *etc.* must be developed.

Therefore, in this article, electromechanical modelling will be performed on a 2-piece system equipped with a multi-flexing technique and a signal processing and control algorithm design based on that. In Section 2, the resonator motion equation with continuous harmonic excitation will be deduced and the major electromechanical gains between the resonator and the electrodes calculated through the modeling. At this time, the mode shape of the resonator will be considered. In addition, the equation of the resonator motion for case that the switched harmonic excitation is applied by the multi-flexing will be induced, and its results will be compared with the case of continuous harmonic excitation. In Section 3, the signal processing and control algorithm will be designed based on the electromechanical modelling results. In this section, the signal processing algorithm based on the multi-flexing method and the differential control algorithm in the rate gyro mode (or FTR mode) will be designed. The designed control algorithm will be tested through Matlab/Simulink SW and the design results will be verified finally by comparing the results of an actually made sensor with the simulation results through suitable experiments.

## 2. Electromechanical Modeling of a HRG with Switched Harmonic Excitations

As mentioned in the previous section, a HRG is a sensor to measure the input angular velocity using the precession motion of the elastic standing wave caused by a Coriolis force. The circular section of the hemispherical resonator in the non-vibration state repeats the circle, horizontal elliptical shape, circle and vertical elliptical shape in the secondary mode [6]. The location of the maximum amplitude and the non-vibration location are referred to as the antinode and node, respectively, and generally, the equation of HRG motion is induced through the secondary resonance mode having two nodes and antinodes [7]. Basically, such a HRG system can be modeled with the secondary spring damper system and due to causes such as mass unbalance, *etc.* anisoelasticity errors and damping mismatch occur, which cause the frequency and Q-factor to split, respectively, which are the most important causes of errors of HRG sensors [8].

### 2.1. Full Equations of Motion with Harmonic Excitations

The general full equations of motion of a HRG considering the influences of frequency coupling and damping imperfection are as follows [8]:

(1) $$\left[\begin{array}{c} \ddot{x}\\ \ddot{y} \end{array}\right]+\left[\begin{array}{cc} C_{11} & C_{12}-2k\Omega \\ C_{21}+2k\Omega & C_{22} \end{array}\right]\left[\begin{array}{c} \dot{x}\\ \dot{y} \end{array}\right]+\left[\begin{array}{cc} K_{11} & K_{12}\\ K_{21} & K_{22} \end{array}\right]\left[\begin{array}{c} x\\ y \end{array}\right]=\left[\begin{array}{c} f_{x}\\ f_{y} \end{array}\right]$$

where Ω is the angular velocity of system about the vertical axis of x-axis and y-aixs, ω is the mean resonant frequency, k is the Brian coefficient (~0.3), *C*_11_ = (2/τ) + Δ(1/τ)cos2*θ_τ_*, *C*_12_ = *C*_21_ = Δ(1/τ)sin2*θ_τ_*, *C*_22_ = (2/τ) − Δ(1/*τ*)cos2*θ_τ_*, *K*_11_ = *ω*^2^ − *ω*Δ*ω*cos2*θ_ω_*, *K*_12_ = *K*_21_ = − *ω*Δ*ω*sin2*θ_ω_*, *K*_22_ = *ω*^2^ + *ω*Δ*ω*cos2*θ_ω_*, *ω*^2^ = (*ω*^2^_1_ + *ω*^2^_2_)/2, 1/*τ* = ½(1/*τ*_1_+1/*τ*_2_), *ω*Δ*ω* = (*ω*^2^_1_ − *ω*^2^_2_)/2, Δ(1/*τ*) = (1/*τ*_1_) − (1/*τ*_2_), $\theta_{\omega }$ is the angle of the unbalance between the x-axis and the major axis of resonance mode, $\theta_{\tau }$ is the angle between the x-axis and the major axis of the linear damper (readers should refer to Appendix A for further details).

To calculate the nominal amplitude and the time constant of each axis, let’s assume that there is no frequency coupling by the damping mismatch and the mass unbalance in the above equation and no input angular velocity. Then, the motion characteristics for the each axis in the above equation can be interpreted as the equation of motion of the secondary spring damper system below:

(2) $$\ddot{x}+\frac{2}{\tau_{n}}\dot{x}+\omega^{2}x=f_{x}=\frac{f}{m_{n}},x\left(0\right)=\dot{x}\left(0\right)=0$$

where $m_{n}$ is modal mass, *τ_n_* = 2Q/*ω*, Q is the quality factor. In this moment, the excitation force to be applied continuously can be modeled with the harmonic function as follows:

(3) $$f\left(t\right)=\frac{df}{dv}v_{c}\left(t\right)$$

where $\frac{df}{dv}$ is the change of control force per unit voltage, $v_{c}\left(t\right)=\bar{v_{c}}\cos\omega t$ is ac control voltage.

**Figure 1:**
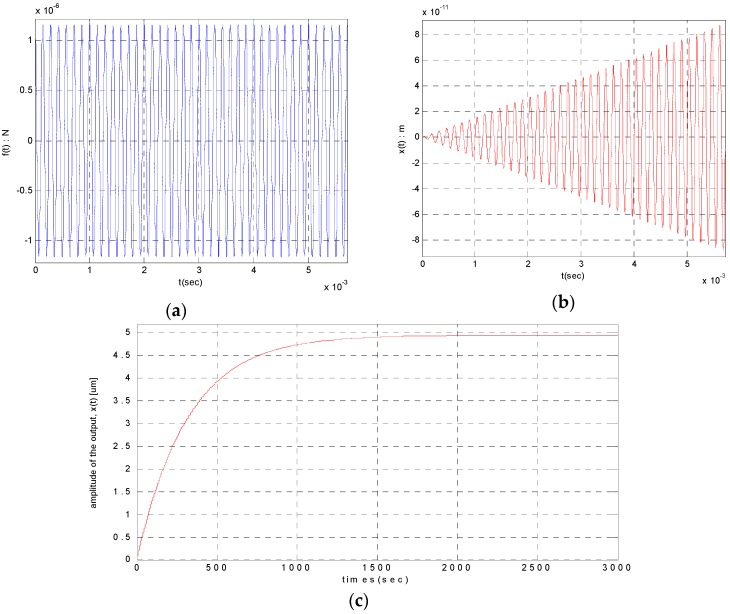
The displacement output by continuous harmonic excitation: (**a**) The control force input (N); (**b**) The displacement output ($t\leq 40\cdot \frac{2\pi }{\omega }\;s$); (**c**) The displacement output (t≤3000 s).

With the aid of Euler’s formula, the solution, x(t) can be written in the form [9]:

(4) $$x\left(t\right)=f_{0}\frac{Q}{m_{n}\omega^{2}}\left(\sin\omega t-\frac{1}{\sqrt{1-{\left(\frac{1}{2Q}\right)}^{2}}}e^{-\frac{\omega }{2Q}t}\sin\omega_{d}t\right)$$

where $f_{0}=\frac{dC}{dx}V_{B}\bar{v_{c}}$, $V_{B}$ is bias voltage, $\frac{dC}{dx}$ is the change of capacitance per unit displacement, $\omega_{d}=\omega \sqrt{1-{\left(\frac{1}{2Q}\right)}^{2}}$. Since $\omega_{d}≅\omega$ with the assumption that the quality factor is high, the equation above can be arranged as follows. The variables needed for deriving the equation are summarized in Table 1 and it can be schematized as shown in Figure 1.

(5) $$x\left(t\right)=f_{0}\frac{Q}{m_{n}\omega^{2}}\sin\omega t\left(1-\frac{1}{\sqrt{1-{\left(\frac{1}{2Q}\right)}^{2}}}e^{-\frac{\omega }{2Q}t}\right)$$

In Equation (5), as $\frac{1}{\sqrt{1-{\left(1/2Q\right)}^{2}}}≅1$ can be assumed, when expressing the amplitude as x(t)=A(t)sin(ωt+ϕ), $A\left(t\right)=A_{n}\left(1-e^{-t/\tau_{n}}\right)$, the nominal amplitude $A_{n}$ and the time constant $\tau_{n}$ can be calculated as follows:

(6) $$A_{n}=\frac{Q}{\pi C_{1}}\left(e^{\frac{\pi C_{2}}{Q}}-1\right)\frac{f_{0}Q}{m_{n}\omega^{2}}=4.971\;\mu m$$

(7) $$\tau_{n}=\frac{2Q}{\omega }=318.310\;s$$

### 2.2. The Nominal Amplitude and Time Constant of HRG with Switched Harmonic Excitations

In the case of a 2-piece HRG system, it switches the sensing and driving cycles and the x-axis and y-axis using the common element, so the excitation force in Equation (3) is not given continuously but as much as the time determined within the certain cycle, which can be expressed as Equation (8) as follows:

(8) $$f\left(t\right)=\{\begin{array}{c} \frac{df}{dv}V_{B}\bar{v_{c}}\cos\omega t\;C_{1}T_{0}\left(n-1\right)<t\leq C_{2}T_{0}+C_{1}T_{0}\left(n-1\right),\;n=1,2,\cdots ,\infty \\ 0\;C_{2}T_{0}+C_{1}T_{0}\left(n-1\right)<t\leq C_{2}T_{0}n,\;n=1,2,\cdots ,\infty \end{array}$$

where $T_{0}=\frac{2\pi }{\omega }s$, $C_{1}=5$ is the total operation cycles, including sensing and driving cycles, $C_{2}=1$ is the x-axis control cycle.

To obtain the system time response characteristics when the non-continuous excitation force is given, the Duhamel integral (or convolution integral) method is used [9]. The Duhamel integral method is the special form of integral to be applied when obtaining the output signal in a linear system if the input signal and the system impulse response are given:

(9) $$x\left(t\right)=f\left(t\right)*h\left(t\right)=\int_{-\infty }^{\infty }f\left(\tau \right)h\left(t-\tau \right)d\tau =\int_{-\infty }^{\infty }h\left(\tau \right)f\left(t-\tau \right)d\tau$$

We get the unit impulse response of a viscously damped SDOF system. By convention, the unit impulse response function is frequently called h(t) [9]:

(10) $$h\left(t-\tau \right)=\frac{1}{m_{n}\omega }e^{-\frac{\omega }{2Q}\left(t-\tau \right)}\sin\omega_{d}\left(t-\tau \right)\text{ at }t\geq \tau$$

**Figure 2:**
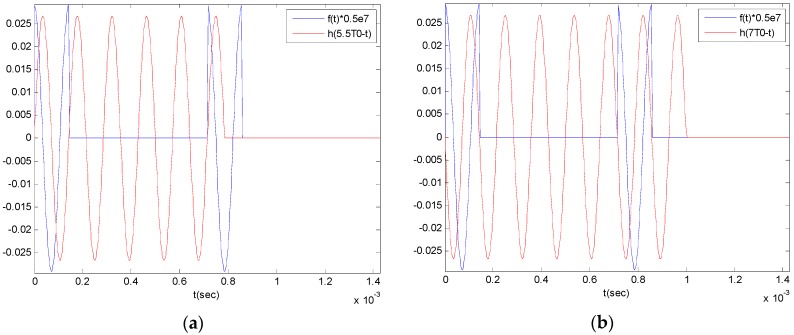
Comparison of the switched control force f(τ) and impulse response h(t−τ): (**a**) case of $C_{1}T_{0}<t\leq C_{2}T_{0}$; (**b**) case of $C_{2}T_{0}+C_{1}T_{0}<t\leq 2C_{1}T_{0}$.

Let’s calculate x(t) by inserting above Equations (8) and (10) into Equation (9). Since f(t)=0 when t<0, x(t)=0. In the section of $C_{1}T_{0}\left(n-1\right)<t\leq C_{2}T_{0}+C_{1}T_{0}\left(n-1\right)$, n=1,2,⋯,∞, as the excitation and the unit impulse response show the phase difference as Figure 2a, and in the section of $C_{2}T_{0}\left(n-1\right)+C_{1}T_{0}<t\leq 2C_{1}T_{0}n$, n=1,2,⋯,∞, they show the phase difference as Figure 2b, the integral can be arranged as follows:

Case a. $C_{1}T_{0}\left(n-1\right)<t\leq C_{2}T_{0}+C_{1}T_{0}\left(n-1\right),n=1,2,\cdots ,\infty$
  (11) $$x\left(t\right)=\overset{k=n-1}{\underset{k=1}{\sum }}\left[\int_{C_{1}T_{0}\left(k-1\right)}^{C_{2}T_{0}+C_{1}T_{0}\left(k-1\right)}f\left(\tau \right)h\left(t-\tau \right)d\tau \right]+\int_{C_{1}T_{0}\left(n-1\right)}^{t}f\left(\tau \right)h\left(t-\tau \right)d\tau$$
Case b. $C_{2}T_{0}\left(n-1\right)+C_{1}T_{0}<t\leq 2C_{1}T_{0}n,n=1,2,\cdots ,\infty$
  (12) $$x\left(t\right)=\overset{k=n}{\underset{k=1}{\sum }}\left[\int_{C_{1}T_{0}\left(k-1\right)}^{C_{2}T_{0}+C_{1}T_{0}\left(k-1\right)}f\left(\tau \right)h\left(t-\tau \right)d\tau \right]$$

Assuming that $x_{ct}\left(t\right)=\int_{C_{1}T_{0}\left(n-1\right)}^{t}f\left(\tau \right)h\left(t-\tau \right)d\tau$, $x_{pt}\left(t\right)=\int_{C_{1}T_{0}\left(k-1\right)}^{C_{2}T_{0}+C_{1}T_{0}\left(k-1\right)}f\left(\tau \right)h\left(t-\tau \right)d\tau$, the calculation results are as follows: as in this moment, the Q-factor is high, we can assume that $\omega_{d}≅\omega$:

(13) $$x_{ct}\left(t\right)=\frac{f_{0}}{m_{n}\omega_{d}}e^{-\frac{\omega }{2Q}t}\int_{C_{1}T_{0}\left(n-1\right)}^{t}\cos\omega \tau \;e^{\frac{\omega }{2Q}\tau }\sin\omega_{d}\left(t-\tau \right)d\tau$$

it can be arranged as follows:

(14) $$x_{ct}\left(t\right)=\frac{f_{0}/m_{n}\omega_{d}}{{\left(\frac{\omega }{2Q}\right)}^{2}+4\omega^{2}}\left[\left(4\omega Q\sin\omega t+\omega \cos\omega t\right)\left(1-e^{\frac{2C_{1}\pi \left(n-1\right)-\omega t}{2Q}}\right)-\frac{\omega }{2Q}\sin\omega t\;e^{\frac{2C_{1}\pi \left(n-1\right)-\omega t}{2Q}}\right]$$

(15) $$x_{pt}\left(t\right)=\frac{f_{0}/m_{n}\omega_{d}}{{\left(\frac{\omega }{2Q}\right)}^{2}+4\omega^{2}}e^{\frac{\pi C_{1}\left(k-1\right)}{Q}}\left(e^{\frac{2\pi C_{2}-\omega t}{2Q}}-e^{-\frac{\omega t}{2Q}}\right)\left[\left(\frac{\omega }{2Q}+4\omega Q\right)\sin\omega_{d}t+\omega \cos\omega_{d}t\right]$$

Refer to Appendix B for further details. If Equations (14) and (15) are inserted to Equations (11) and (12), the time response of SDOF spring-damper system by the switched excitation can be arranged as follows:

Case a. $C_{1}T_{0}\left(n-1\right)<t\leq C_{2}T_{0}+C_{1}T_{0}\left(n-1\right),n=1,2,\cdots ,\infty$
  (16) $$x\left(t\right)=\frac{\frac{f_{0}}{m_{n}\omega_{d}}}{{\left(\frac{\omega }{2Q}\right)}^{2}+4\omega^{2}}\{\overset{k=n-1}{\underset{k=1}{\sum }}e^{\frac{\pi C_{1}\left(k-1\right)}{Q}}\left(e^{\frac{2\pi C_{2}-\omega t}{2Q}}-e^{-\frac{\omega t}{2Q}}\right)\left[\left(\frac{\omega }{2Q}+4\omega Q\right)\sin\omega_{d}t+\omega \cos\omega_{d}t\right]\;+\left[\left(4\omega Q\sin\omega t+\omega \cos\omega t\right)\left(1-e^{\frac{2\pi C_{1}\left(n-1\right)-\omega t}{2Q}}\right)-\frac{\omega }{2Q}\sin\omega t\;e^{\frac{2\pi C_{1}\left(n-1\right)-\omega t}{2Q}}\right]\}$$
Case b. $C_{2}T_{0}\left(n-1\right)+C_{1}T_{0}<t\leq 2C_{1}T_{0}n,n=1,2,\cdots ,\infty$
  (17) $$x\left(t\right)=\frac{\frac{f_{0}}{m_{n}\omega_{d}}}{{\left(\frac{\omega }{2Q}\right)}^{2}+4\omega^{2}}\overset{k=n}{\underset{k=1}{\sum }}e^{\frac{\pi C_{1}\left(k-1\right)}{Q}}\left(e^{\frac{2\pi C_{2}-\omega t}{2Q}}-e^{-\frac{\omega t}{2Q}}\right)\left[\left(\frac{\omega }{2Q}+4\omega Q\right)\sin\omega_{d}t+\omega \cos\omega_{d}t\right]$$

The time response characteristics of the mean amplitude by the switched excitation are as follows:

(18) $$\bar{A}\left(t\right)=\frac{Q}{\pi C_{1}}\left(e^{\frac{\pi C_{2}}{Q}}-1\right)\frac{f_{0}Q}{m_{n}\omega^{2}}\left[1-e^{-\frac{\left(\omega -\frac{C_{1}-C_{2}}{C_{1}}\right)}{2Q}t}\right]$$

Refer to Appendix C for further details. Accordingly, when $C_{1}=5,\;C_{2}=1\;$, the nominal amplitude and the time constant are calculated as follows:

(19) $${\bar{A}}_{n}=\frac{Q}{\pi C_{1}}\left(e^{\frac{\pi C_{2}}{Q}}-1\right)\frac{f_{0}Q}{m_{n}\omega^{2}}≅0.2\frac{f_{0}Q}{m_{n}\omega^{2}}=0.994\;\mu m$$

(20) $${\bar{\tau }}_{n}=\frac{2Q}{\omega -\frac{C_{1}-C_{2}}{C_{1}}}=318.316\;s$$

Through the above results, it is observed that the amplitude can be obtained by multiplying the rate of the driving cycle against the entire operation cycle compared with the amplitude when the continuous excitation is applied, and the time constants are almost same.

**Figure 3:**
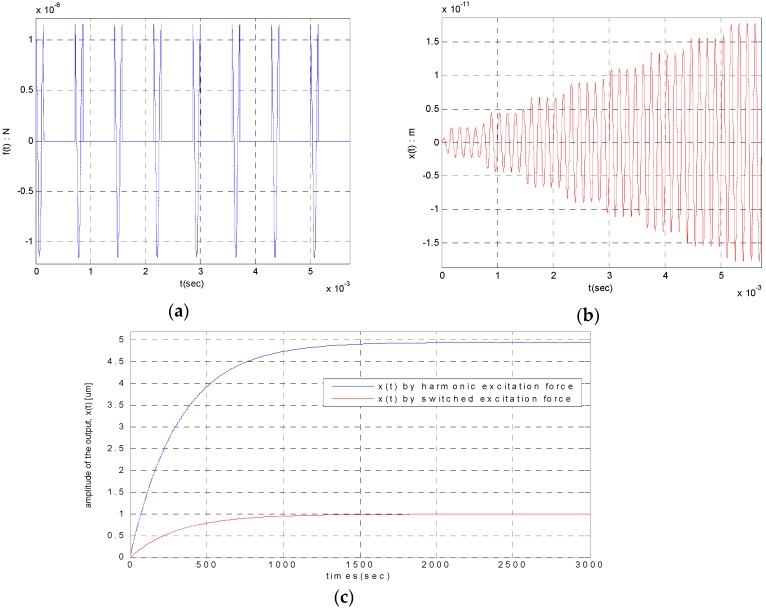
The displacement output by switched harmonic excitation: (**a**) The control force input (N); (**b**) The displacement output ($t\leq 40\cdot \frac{2\pi }{\omega }\;s$); (**c**) The displacement output (t≤3000 s).

This is the important input data for electromechanical gains and design of controller. The amplitude results are schematized as shown in Figure 3.

### 2.3. Verification of the Analytic Results through Simulations

**Figure 4:**
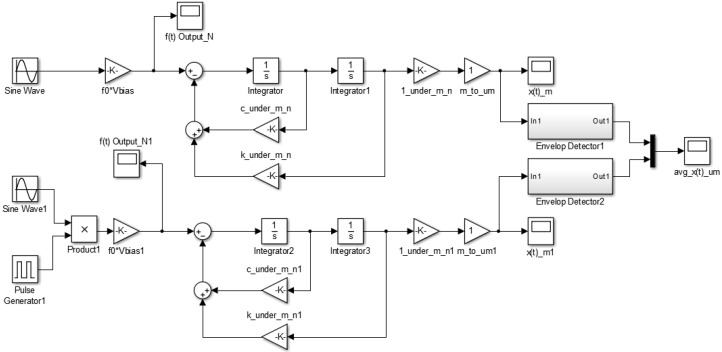
Simulation SW to verify the analytic results of the time response by switched excitation.

**Figure 5:**
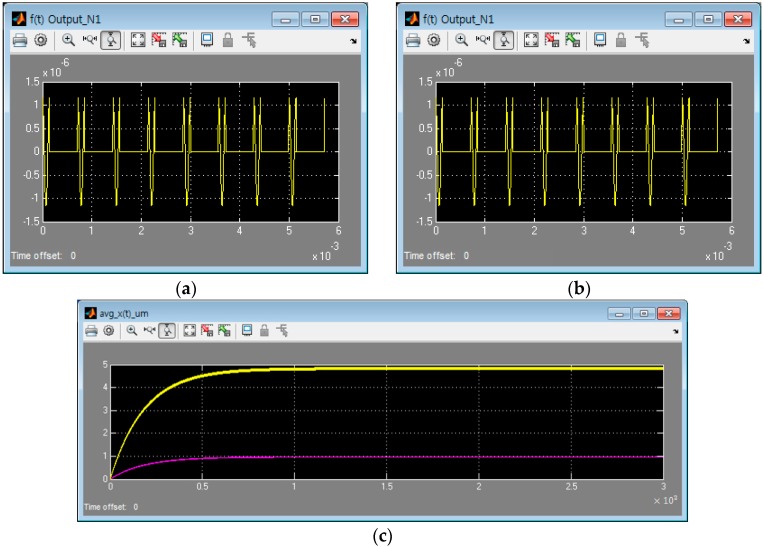
The displacement output by switched harmonic excitation [Matlab/Simulink]: (**a**) The control force input (N); (**b**) The displacement output ($t\leq 40\cdot \frac{2\pi }{\omega }\;s$); (**c**) The displacement output (t≤3000 s) (yellow: continuous/pink: switched excitation).

To test the above interpretation results, the Matlab/Simulink SW was prepared as shown in Figure 4 and the simulation was performed. It is observed that the results are as same as Figure 5 and is identical when comparing with Figure 3.

### 2.4. Electromechanical Modeling between Resonator and Electrodes

Before designing the signal processing and control circuit, the electromechanical modeling between resonator and electrodes must be done to estimate the capacitance change for different electrostatic forces.

The gap between the resonator and the sensing (or driving) electrode block cannot be assumed as parallel plate simply. For the resonator, the 2nd vibration shape corresponding to a standing wave in the thin hemispherical shell should be considered. Therefore, in this article, the capacitance change and the changes in the electrostatic force are calculated considering the mode shape and modal force.

**Figure 6:**
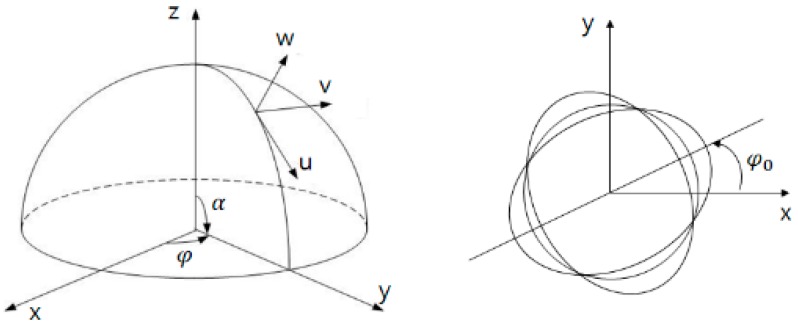
The position of a point on the shell with the angular coordinates α,φ in a Cartesian coordinate frame fixed to the resonator.

According to Rayleigh, the mode equation for the mode shape analysis among the second vibration mode equations of the thin hemispherical shell of the resonator can be represented [10]:

(21) $$w=\frac{A}{2}\left(2+\cos\alpha \right)\tan^{2}\frac{\alpha }{2}\cos\left[2\left(\varphi -\varphi_{0}\right)\right]\sin\left[\omega \left(t-t_{0}\right)\right]+\frac{B}{2}\left(2+\cos\alpha \right)\tan^{2}\frac{\alpha }{2}\sin\left[2\left(\varphi -\varphi_{0}\right)\right]\cos\left[\omega \left(t-t_{0}\right)\right]$$

where A,B is the 1st and 2nd wave amplitude, $\varphi_{0}$ is the orientation of the wave relative to the resonator. w,α,φ is clear from Figure 6.

The mode shape calculated by inserting $\varphi_{0}=0,\;A=1,\;B=0$ to Equation (21) is as follows:

(22) $$\phi \left(\alpha ,\;\varphi \right)=\;\frac{1}{2}\left(2+\cos\alpha \right)\tan^{2}\frac{\alpha }{2}\cos2\varphi$$

The electrostatic capacitance $C_{0}$ and capacitance changes by the displacement $\frac{dC}{dx}$ in the sensing electrode are induced as follows using above equation:

(23) $$C_{0}=\int_{\alpha_{1}}^{\alpha_{2}}\int_{\varphi_{1}}^{\varphi_{2}}\frac{\varepsilon {\left(R-d_{0}\right)}^{2}}{d_{0}}d\alpha d\varphi =\frac{\varepsilon {\left(R-d_{0}\right)}^{2}}{d_{0}}\left(\alpha_{2}-\alpha_{1}\right)\left(\varphi_{2}-\varphi_{1}\right)$$

(24) $$\frac{dC}{dx}=\int_{\alpha_{1}}^{\alpha_{2}}\int_{\varphi_{1}}^{\varphi_{2}}-\frac{\varepsilon {\left(R-d_{0}\right)}^{2}\phi \left(\alpha ,\;\varphi \right)}{{\left[d_{0}+q\left(t\right)\phi \left(\alpha ,\;\varphi \right)\right]}^{2}}d\varphi d\alpha \cdot \frac{\left(\alpha_{2}-\alpha_{1}\right)+\left(\varphi_{2}-\varphi_{1}\right)}{4\pi }$$

where $x\left(t\right)=d_{0}+q\left(t\right)\phi \left(\alpha ,\;\varphi \right)$, q(t) is a modal amplitude. See Table 2 for the rest of the variables. If Equation (22) is inserted to Equation (24), it is as follows:

(25) $$\frac{dC}{dx}≅-\frac{\varepsilon {\left(R-d_{0}\right)}^{2}}{2d_{0}^{2}}\cdot \frac{\left(\alpha_{2}-\alpha_{1}\right)+\left(\varphi_{2}-\varphi_{1}\right)}{4\pi }\int_{\alpha_{1}}^{\alpha_{2}}\left(2+\cos\alpha \right)\tan^{2}\frac{\alpha }{2}d\alpha \int_{\varphi_{1}}^{\varphi_{2}}\cos2\varphi d\varphi$$

(26) $$\int_{\varphi_{1}}^{\varphi_{2}}\cos2\varphi d\varphi =\frac{1}{2}{\left[\sin2\varphi \right]}_{\varphi_{1}}^{\varphi_{2}}$$

(27) $$\int_{\alpha_{1}}^{\alpha_{2}}\left(2+\cos\alpha \right)\tan^{2}\frac{\alpha }{2}d\alpha ={\left[4\tan\frac{\alpha }{2}-2\tan\alpha -\frac{2}{\sin\alpha }-\sin\alpha \right]}_{\alpha_{1}}^{\alpha_{2}}$$

If Equations (26) and (27) is inserted to Equation (25), it is as follows:

(28) $$\frac{dC}{dx}≅-\frac{\varepsilon {\left(R-d_{0}\right)}^{2}}{4d_{0}^{2}}\cdot \frac{\left(\alpha_{2}-\alpha_{1}\right)+\left(\varphi_{2}-\varphi_{1}\right)}{4\pi }{\left[\sin2\varphi \right]}_{\varphi_{1}}^{\varphi_{2}}{\left[4\tan\frac{\alpha }{2}-2\tan\alpha -\frac{2}{\sin\alpha }-\sin\alpha \right]}_{\alpha_{1}}^{\alpha_{2}}$$

The calculating the control force f by the control voltage $v_{ac}$ applied to the electrode block is as follows:

(29) $$\frac{df}{dv_{ac}}≅\frac{\varepsilon {\left(R-d_{0}\right)}^{2}\left(V_{bias}+v_{ac}\right)}{4d_{0}^{2}}\cdot \frac{\left(\alpha_{2}-\alpha_{1}\right)+\left(\varphi_{2}-\varphi_{1}\right)}{4\pi }\int_{\alpha_{1}}^{\alpha_{2}}\left(2+\cos\alpha \right)\tan^{2}\frac{\alpha }{2}d\alpha \int_{\varphi_{1}}^{\varphi_{2}}\cos2\varphi d\varphi$$

Equations (26) and (27) are inserted to Equation (29), it can be arranged as follows:

(30) $$\frac{df}{dv_{ac}}≅\frac{\varepsilon {\left(R-d_{0}\right)}^{2}\left(V_{bias}+v_{ac}\right)}{4d_{0}^{2}}\cdot \frac{\left(\alpha_{2}-\alpha_{1}\right)+\left(\varphi_{2}-\varphi_{1}\right)}{4\pi }{\left[\sin2\varphi \right]}_{\varphi_{1}}^{\varphi_{2}}{\left[4\tan\frac{\alpha }{2}-2\tan\alpha -\frac{2}{\sin\alpha }-\sin\alpha \right]}_{\alpha_{1}}^{\alpha_{2}}$$

The equation related to the amplitude of resonator by the control force is as follows:

(31) $$\frac{dx}{df}=\frac{Q}{m_{n}\omega^{2}}$$

The equation of the change amplifier output by the change in the amplitude is as follows:

(32) $$\frac{dv_{ca}}{dC}=-\frac{1}{C_{f}}V_{bias}$$

By inserting the values in Table 2 into Equations (23), (28), (30)–(32), the electromechanical gains in Table 3 can be obtained:

If 5-cycles operation multi-flexing method, where $C_{1}=5\;,\;C_{2}=1$, is applied and four electrode blocks are assigned to the signals of x-axis and y-axis, the control force, the amplitude and the output voltage of charge amplifier by control voltage of 100 mVac are follows:

(33) $${f|}_{v_{ac}=100mV}=\frac{1}{5}\times 2.78\times 10^{-6}\times 4\times 100\times 10^{-3}=2.224\times 10^{-7}\;\left(N\right)$$

(34) $${x|}_{v_{ac}=100mV}=2.224\times 10^{-7}\times 4.26=0.9474\;\left(\mu m\right)$$

(35) $${v_{ca}|}_{v_{ac}=100mV}=9.1\times 10^{12}\times 17.5\times 10^{-9}\times 0.9474\times 10^{-6}=150.7\;\left(\mathrm{mV}\right)$$

In the measurement results after making actual sensor, the amplitude by the control voltage of 100 mV was 1.02 μm and the output voltage of the charge amplifier was 143 mV showing 5.1% and 6.9% of differences, respectively, which proves that the design results are valid when considering the error in the process and measurement. Based on the electromechanical gains mentioned above, the proper signal processing and control circuit will be designed.

## 3. Design of the Signal Processing and Control Algorithm

A HRG can be operated as an angular velocity sensor or an angular sensor with a single sensor without structural change. In this study, FTR mode (or rate gyro mode), which has dynamic range limitations but excellent noise and resolution characteristics, will be handled. FTR mode is driven by the closed loop. In the FTR mode, the driving-axis is excited to remain as the reference amplitude and the sensing-axis generated by input of angular velocity controls the amplitude to be 0. In this moment, the force required to remove the sensing-axis vibration is referred to as the rebalance force and as it is proportional to the angular velocity input, the angular velocity input is estimated by multiplying this force by the conversion scale factor [11].

**Figure 7:**
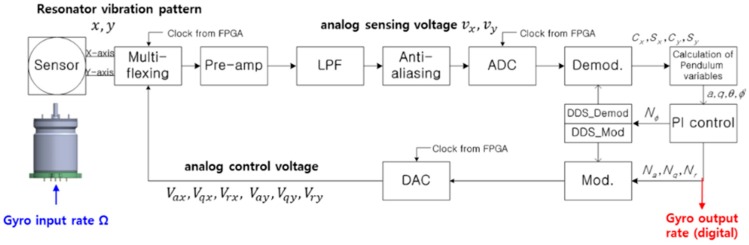
HRG signal processing and control circuit block diagram.

As shown in Figure 7, if the analog voltage output comes out from the pre-amplifier, it is transmitted to the FPGA in the form of a digital signal through the filter and ADC. It is converted to the in-phase, quadrature signals of x- and y-axis through the demodulation in the FPGA. These signals are calculated from the pendulum variables required for control and digital PI control outputs for amplitude, quadrature, rate and phase control are generated in the DSP. If the control outputs are delivered to the FPGA, the driving signal is generated by the modulation, which becomes an analog control voltage through the DAC controlling the HRG. In addition, in the DSP, it was designed to output the angular velocity by estimating the input angular velocity with rate control output.

### 3.1. Design of the Signal Processing Algorithm

**Figure 8:**
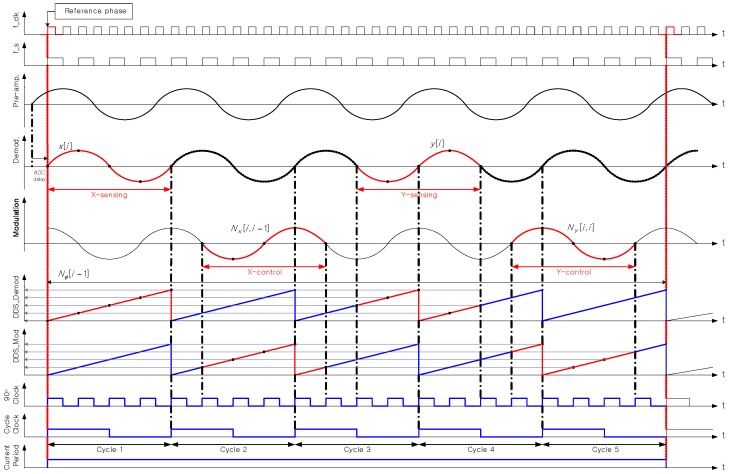
HRG sensing and control signal time diagram.

In this study, the ADC sampling frequency $f_{s}$ was designed with 512 times the resonance frequency. If the FPGA clock frequency $f_{clk}$ is assumed as $2f_{s}$ for the convenience and the sensing and control timing diagram is schematized as shown in Figure 8 and Table 4. In the actual algorithm, $f_{clk}=n_{s}f_{s}$ ($n_{s}$ is an integer). The reference phase ϕ is assumed to be 0, which means that sinωt,cosωt are used for the demodulation signal. In the time diagram, [i] indicates i-th operation period. [i-1] indicates previous operation period. If x(t) is expressed as $c_{x}\cos\omega t+s_{x}\sin\omega t$ and y(t) is expressed as $c_{y}\cos\omega t+s_{y}\sin\omega t$, Direct Digital Synthesis (DDS) is used to obtain $c_{x},s_{x},c_{y}\text{ and }s_{y}$. As the phase of DDS and the demodulation reference phase are same, the cosine and sine output of the DDS is multiplied by x(t) as is. As y(t) is reversed in the signal intended for demodulation originally and the phase of DDS has difference of π (180°) from the demodulation reference phase, it is multiplied to y(t) as is like x(t).

Since x(t) and y(t) are measured discontinuously by the multi-flexing, $c_{x},s_{x},c_{y}\text{ and }s_{y}$ cannot be calculated using conventional LPF. Therefore they will be calculated averaging the sensing signal as in the equations below:

(36) $$c_{x}\approx \frac{2}{N_{s}}\overset{N_{s}}{\underset{i=1}{\sum }}x\left(t_{i}\right)\cos\omega t_{i},\;s_{x}\approx \frac{2}{N_{s}}\overset{N_{s}}{\underset{i=1}{\sum }}x\left(t_{i}\right)\sin\omega t_{i}$$

(37) $$c_{y}\approx \frac{2}{N_{s}}\overset{N_{s}}{\underset{i=1}{\sum }}y\left(t_{i}\right)\cos\omega t_{i},\;s_{y}\approx \frac{2}{N_{s}}\overset{N_{s}}{\underset{i=1}{\sum }}y\left(t_{i}\right)\sin\omega t_{i}$$

where $N_{s}$ is the number of FPGA clocks for sampling cycle (= 512).

**Figure 9:**
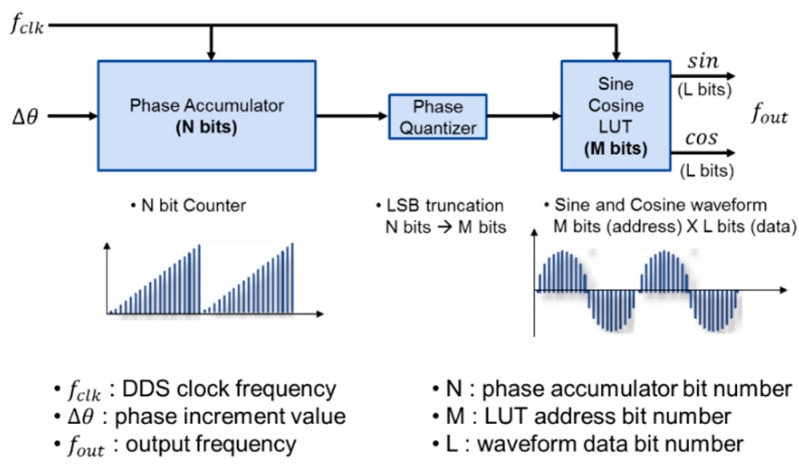
The structure of the DDS (Direct Digital Synthesis).

It was designed that the phase delay $\phi_{corr}$ of the electronic circuit is compensated in the modulation stage of control command calculated through the PI controller. DDS_Mod cosine and sine outputs correspond to $\cos\left(\omega t+\phi_{corr}\right)$ and $-\sin\left(\omega t+\phi_{corr}\right)$, respectively. The internal frequency of FPGA is generated in the Numerical Controlled Oscillator (NCO). This NCO and the block including the sine and cosine wave Look-Up Table (LUT) are referred to as the DDS. DDS, which generates the signal required for demodulation and modulation, is composed of phase accumulator, phase quantizer and LUT as shown in Figure 9. In Figure 9, the equation related of DDS clock frequency $f_{clk}$, accumulator bit number N and phase increment value Δθ and the calculus of the DDS frequency resolution Δf are as follows:

(38) $$f_{out}=\frac{f_{clk}\Delta \theta }{2^{N}}Hz,\Delta f=\frac{f_{clk}}{2^{N}}Hz$$

The data stored in LUT are $\sin\left(n\frac{2\pi }{2^{M}}\right)$ and $\cos\left(n\frac{2\pi }{2^{M}}\right)$ and n is the integer satisfying $n\in \left[0,\;2^{M}-1\right]$. For DDS output for demodulation, the phase offset is applied and it was designed to add $N_{corr}$ obtained by below acquisition equation to the address bit number M:

(39) $$N_{corr}=\frac{2^{M}}{2\pi }\times \theta_{corr}$$

**Figure 10:**
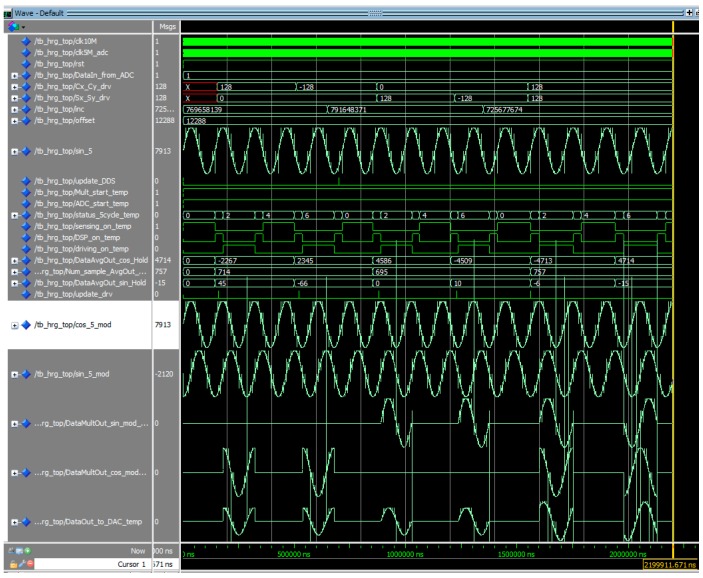
Total operation simulation result of the FPGA signal processing algorithm.

Figure 10 shows the entire motion simulation results of FPGA signal processing algorithm explained so far.

### 3.2. Design of the Control Algorithm

The control algorithm is designed to pursue the ideal gyro motion without flaw. In this design, the definition of IEEE Std 1431 Annex B (D. Lynch) is used [12]. In the case of a shell resonator, which has no damping and the elasticity is axially symmetrical, since E and Q are not changed regardless of pattern angle θ and orbit phase $\phi^{\prime }$, these two invariants are used as amplitude and quadrature control variable, respectively. In the FTR mode, S and Q are used as rate and phase control variable, respectively. The control variables are shown in Table 5, the control commands are PI controller outputs and the unit is bits.

The relation between the demodulated signal ($c_{x},\;s_{x},\;c_{y},\;s_{y}$) and the control variables in Table 5 is follows [12]:

(40) $$c_{x}^{2}+s_{x}^{2}+c_{y}^{2}+s_{y}^{2}=a^{2}+q^{2}\equiv E$$

(41) $$2\left(c_{x}s_{y}-c_{y}s_{x}\right)=2aq\equiv Q$$

(42) $$c_{x}^{2}+s_{x}^{2}-c_{y}^{2}-s_{y}^{2}=\left(a^{2}-q^{2}\right)\cos2\theta \equiv R$$

(43) $$2\left(c_{x}c_{y}+s_{x}s_{y}\right)=\left(a^{2}-q^{2}\right)\sin2\theta \equiv S$$

(44) $$2\left(c_{x}s_{x}+c_{y}s_{y}\right)=-\left(a^{2}-q^{2}\right)\sin2\phi^{\prime }\equiv L$$

(45) $$\theta =\frac{1}{2}\tan^{-1}\frac{S}{R}$$

(46) $$\phi^{\prime }=-\frac{1}{2}\sin^{-1}\frac{L}{\sqrt{E^{2}-Q^{2}}}$$

**Figure 11:**
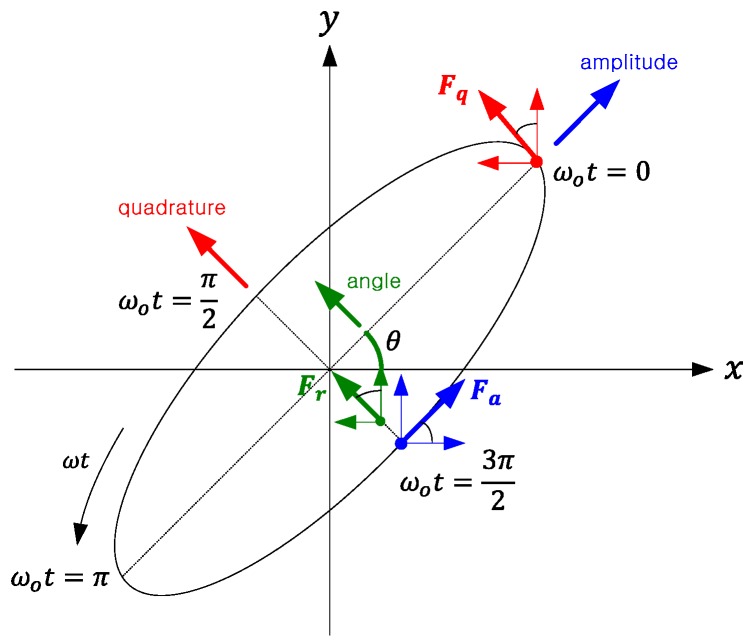
Components of the control forces in the orbit trajectory.

As above, if the control variables are calculated, the resonant frequency must be sought through PLL and the amplitude, quadrature and rate digital control force must be calculated through PI control. The calculation results by referring to Figure 11 and Table 6 are Equations (47) and (48). When the DAC scale factor is $k_{DA}$(V/bit) and the voltage-to-force scale factor is $k_{fc}$(force/volt.), $F_{x}=k_{fc}k_{DA}N_{x}$, $F_{y}=k_{fc}k_{DA}N_{y}$.

(47) $$N_{x}\left(t\right)=-\left(N_{a}\cos\theta -N_{r}\sin\theta \right)\sin\left(\omega t+\phi_{corr}\right)-N_{q}\sin\theta \cos\left(\omega t+\phi_{corr}\right)$$

(48) $$N_{x}\left(t\right)=-\left(N_{a}\sin\theta +N_{r}\cos\theta \right)\sin\left(\omega t+\phi_{corr}\right)+N_{q}\cos\theta \cos\left(\omega t+\phi_{corr}\right)$$

The error for PI control is calculated as shown in Table 7. For integral, the trapezoidal rule is used. It should be noted that for the phase control, the frequency input must be reduced when the phase error is negative. For improving stability and compensating the truncation error, the limiter and summation block are applied to the controller.

### 3.3. Numerical Verification of the Algorithm through Simulations

**Figure 12:**
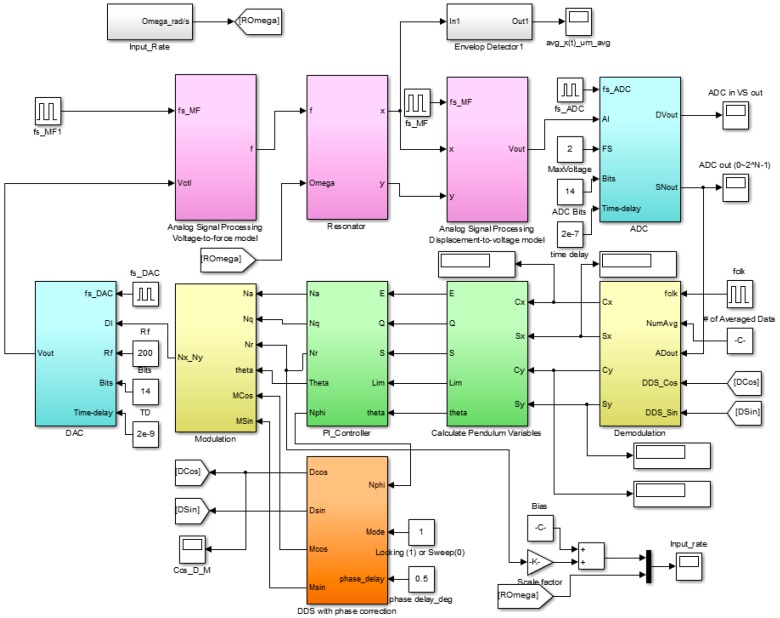
Matlab/Simulink simulation program to verify the signal processing and control algorithm.

Based on the design results of Section 2 and Section 3, the Matlab/Simulink simulation SW is employed as shown in Figure 12. The target bandwidth by control loop is as shown in Table 8. The process of control variables is approximated by a first- or second-order plus delay model (E & S: first-order, Q & L: second-order). The controller tunings are based on these models by using the recommended SIMC-PID method [13]. Table 9 shows the comparison between the control gains satisfying the bandwidth in the simulation program and the control gains in the actual DSP.

**Figure 13:**
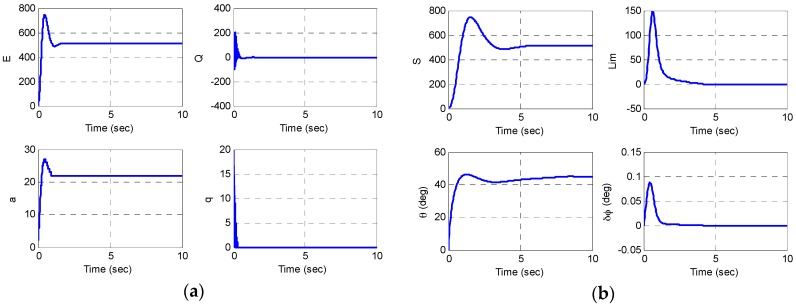
Matlab/Simulink simulation results; control varialbes: (**a**) the amplitude and quadrature control variables; (**b**) the rate and phase control variables.

**Figure 14:**
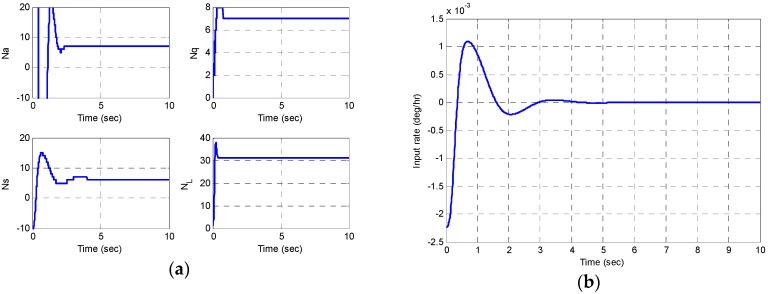
Matlab/Simulink simulation results: (**a**) the digital optputs to control; (**b**) an estimate of the input rate.

The simulation results are shown in Figure 13 and Figure 14. The amplitude control variable is converged to the target amplitude $E_{0}=512\;\mathrm{bits}\;\left(=1\;{\mu m}^{2}\right)$ and the rate control variable is converged well to the target azimuth angle $\theta_{0}=45{}^{\circ}\;\left(S_{0}=512\text{ bits}\right)$. An estimate of the input rate is obtained by taking the difference of demodulated two forces, while the quadrature is nulled out [13].

### 3.4. Experimental Verification of the Algorithm

**Figure 15:**
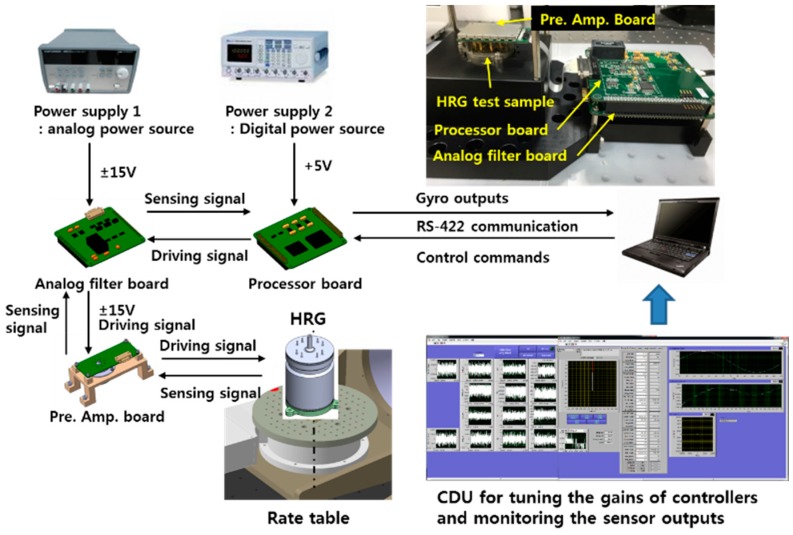
Test set for verification of the signal processing and control algorithm.

**Figure 16:**
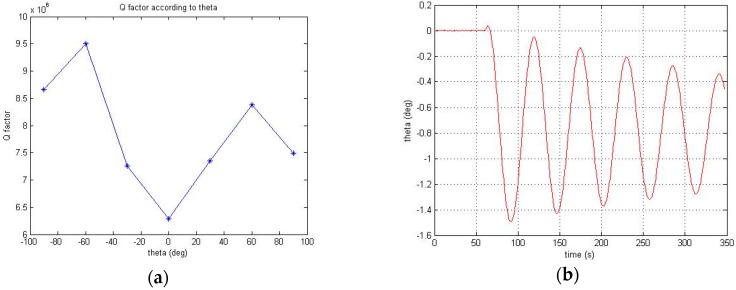
A estimate of the Q, ΔQ and Δf by using the controllers: (**a**) Measured Q-factor in according to target angle θ; (**b**) Vibration of the target angle θ.

Figure 15 shows the test set to test the design and to conduct the gyro performance test linking with electronic board equipped with sensor, signal processing and control algorithm. First of all, the gyro parameters (Q, ΔQ, f, Δf) were measured after tuning the PI control gain suitable for sensor. When the control is stabilized, the amplitude control is turned off and the Q-factor is calculated by measuring the time constant by the target azimuth angle *θ*. At this time, ΔQ can be estimated from the difference between the maximum value and the minimum value. The test results are shown in Table 10 and Figure 16a. Then, if only the quadrature and rate control are turned off while maintaining the phase control and the amplitude control, *θ* is vibrated with certain cycle as shown in Figure 16b and this vibration frequency is the resonance frequency split Δf. As shown in Figure 16b, in case of test sample, the vibration cycle is approximately 55 s and the frequency split Δf is approximately 18 mHz.

**Figure 17:**
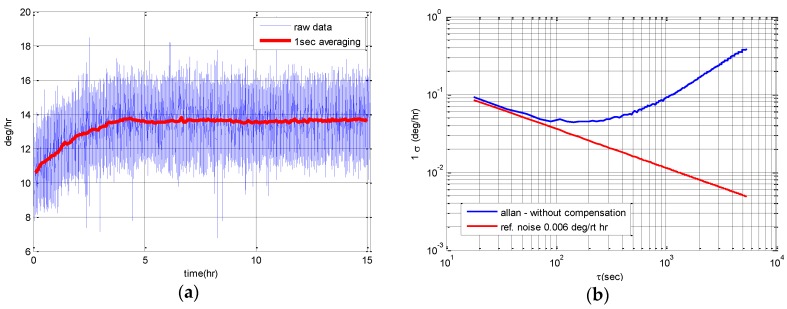
Test results to measure the bias instability and ARW: (**a**) Gyro rate output for 15 h in the sound absorbing chamber; (**b**) Allan variance analysis result for the estimate of bias instability and ARW.

Next, the gyro scale factor and the bias were measured using the rate table and the bias instability and Angle Random Walk (ARW) measuring test were performed within a sound absorbing chamber. Bias instability and ARW are calculated through Allan variance using the data that recorded the gyro output for more than 8 h after stabilizing the gyro in a state isolated from the disturbance. As shown in Figure 17, in case of the sample used in this study, the bias instability is 0.07°/h and ARW is 0.006°/(rt·h).

## 4. Conclusions

HRG is one of the kinds of CVG, which measures the angle or angular velocity using the Coriolis force produced by the rotational motion. The rotational angle or angular velocity can be measured using the principle that the standing wave generated in the quartz resonator in the form of hemisphere shell performs the procession motion.

HRG technology development is underway in many advanced countries and the next generation HRGs, which can materialize the objectives of subminiature size, high precision and high reliability with the 2-piece HRG system is being applied with multi-flexing method and differential control development in the United States, France, *etc.*

Therefore, in this article, a controller design suitable for a 2-piece HRG system was performed. To design the controller, the electromechanical modeling of the 2-piece HRG system was pre-performed. To interpret the vibration characteristics due to the switched discontinuous excitation force, the Duhamel integral method was applied. In addition the electromechanical gains were calculated considering the mode shape of the thin hemispherical shell. It was proven that the design results are valid by showing an error within 7% in the comparison between design results and measurement results of the amplitude and charge amplifier output.

Based on such modeling, the signal processing based on the multi-flexing method and differential control algorithm were designed. The sensing and driving cycles of x- and y-axis were divided by time with five operation cycles using a common element. The sensing signals generate the control inputs signal through the sampling and demodulation processes. The controller output generates the final control voltages through the modulation processes and the phase delay compensation algorithm was applied in the modulation process. In FTR mode, Control is composed of phase, amplitude, quadrature and rate control and the pendulum variables are used as control variables. The designed algorithm was verified through Matlab/Simulink simulation and in the results, the bandwidth of the amplitude and quadrature control were satisfactory, at 1–5 Hz, and the bandwidth of the rate and phase control were satisfactory at 7.5–12.5 Hz. Finally, the electronic circuit was made by equipping the algorithm in FPGA and DSP and that it satisfied with the target bandwidth was verified through the experiment. In addition, through the sensor linking test, the error identification was performed and it was conformed that the bias instability and ARW are approximately 0.07°/h and 0.006°/(rt·h), respectively by performing the gyro performance test.

## Figures and Tables

**Table 1:** The design variables for the full time equation of motion.

Design Variables	Values
Resonant Frequency	ω=7.0 kHz×2π(rad/s)
Quality factor	$Q=7\times 10^{6}$
Modal mass	$m_{n}=0.85\times 10^{-3}\;\mathrm{kg}$
The change of control force per unit voltage	$df/dv=2.78\times 10^{-6}\;N/V\;$
Bias voltage	$V_{B}=200\;V$
Nominal control voltage	$\bar{v_{c}}=420\;\mathrm{mV}$
The change of capacitance per unit displacement	$dC/dx=13.9\times 10^{-9}\;F/m$

**Table 2:** The design variables for calculating the electromechanical gains.

Design Variables	Values
Radius of Resonator	R=15.3mm
Nominal gap between resonator and electrode block	$d_{0}=120\;\mu m$
Dielectric permittivity	$\varepsilon =8.85\times 10^{-12}\;F/m$
Electrodes angles in azimuth	$\varphi_{1}=-18{}^{\circ},\;\varphi_{2}=18{}^{\circ}$
Electrodes angles in elevation	$\alpha_{1}=78.2{}^{\circ},\;\alpha_{2}=90{}^{\circ}$
Bias voltage	$V_{bias}=200\;V$
Quality factor	$Q=7\times 10^{6}$
Modal mass	$m_{n}=0.85\times 10^{-3}\;\mathrm{kg}$
Resonant frequency	$\omega =4.4\times 10^{4}\;\mathrm{rad}/s$
Feedback capacitance of the charge amp	$C_{f}=22\;\mathrm{pF}$

**Table 3:** The electromechanical gains.

	Case of the Parallel Plate Capacitor	Case of Considering the Mode Shape of Resonator
$C_{0}$	3.8 pF	3.8 pF
$\frac{dC}{dx}$	$17.5\times 10^{-9}\;F/m$	$13.9\times 10^{-9}\;F/m$
$\frac{df}{dv_{ac}}$	$3.5\times 10^{-6\;}\;N/V$	$2.78\times 10^{-6}\;N/V$
$\frac{dx}{df}$	4.26 m/N	4.26 m/N
$\frac{dv_{ac}}{dC}$	$9.1\times 10^{12}\;V/F$	$9.1\times 10^{12}\;V/F$

**Table 4:** The update period of control commands.

Control Command	Designation	The Period
$N_{\phi }$	Phase control	1 operation period
$N_{a}$	Amplitude control	X-control: update after X-sensing $N_{x}=f\left(c_{x}\left[i\right],\;s_{x}\left[i\right],c_{y}\left[i-1\right],\;s_{y}\left[i-1\right]\right)$ Y-control: update after Y-sensing $N_{y}=f\left(c_{x}\left[i\right],\;s_{x}\left[i\right],c_{y}\left[i\right],\;s_{y}\left[i\right]\right)$
$N_{q}$	Quadrature control	
$N_{r}$	Rate control	

**Table 5:** The design of control variables.

Control variable	Formula	Designation	Control Command	Target Value
E	$a^{2}+q^{2}$	Amplitude	$N_{a}$	$E_{0}=1\;{\mu m}^{2}\;$
Q	2aq	Quadrature	$N_{q}$	$Q_{0}=0\;{\mu m}^{2}$
S	$\left(a^{2}-q^{2}\right)\sin2\theta$	Rate	$N_{r}$	$S_{0}=1\;{\mu m}^{2}$
L	$-\left(a^{2}-q^{2}\right)\sin2\phi^{\prime }$	Phase	$N_{\phi }$	$L_{0}=0\;{\mu m}^{2}$

**Table 6:** Components of the control forces.

Control Variable	Control Force (Digital Control Command)	Input Phase	$N_{x}$ Component	$N_{y}$ Component
E	$F_{a}\left(N_{a}\right)$	$-\sin\left(\omega t+\phi_{corr}\right)$	$N_{a}\cos\theta$	$N_{a}\sin\theta$
Q	$F_{q}\left(N_{q}\right)$	$\cos\left(\omega t+\phi_{corr}\right)$	$-N_{q}\sin\theta$	$N_{q}\cos\theta$
S	$F_{r}\left(N_{r}\right)$	$-\sin\left(\omega t+\phi_{corr}\right)$	$-N_{r}\sin\theta$	$N_{r}\cos\theta$

**Table 7:** Design of PI Controller.

Control Variable	Error	Command	PI Control Output
*E*	$e_{a}=E_{0}-E$	$N_{a}$	$N_{k}=N_{k-1}+K_{P}\left(e_{k}-e_{k-1}\right)+\frac{T_{s}}{2T_{I}}\left(e_{k}+e_{k-1}\right)$
*Q*	$e_{q}=-Q$	$N_{a}$	where $K_{P}$ is the Proportional gain $T_{I}$ is the Integral time $T_{s}$ is sampling time (=$1/f_{s}$)
*S*	$e_{r}=S_{0}-S$	$N_{a}$	
*L*	$e_{\phi }=L$	$N_{a}$	

**Table 8:** The bandwidth goals of control loop.

Control Loop	Phase (PLL)	Amplitude	Quadrature	Rate
Bandwidth (Hz)	7.5–12.5	1–5	1–5	7.5–12.5

**Table 9:** Comparison of controller gains for simulation and DSP uploading program.

Design Variables of the Controller	Matlab/Simulink Simulation Ver.	DSP Uploading Program Ver.
Frequency	7.1 kHz	7.12605 kHz
Target Amp.($E_{0}$)	512 bits (scaling)	540 bits (scaling)
Phase Delay	1820 bits	1682 bits
Phase control P gain	6000 bits	4000 bits
Phase control I gain	10 bits	25 bits
Amplitude control P gain	725 bits	700 bits
Amplitude control I gain	1 bits	1 bits
Quadrature control P gain	800 bits	700 bits
Quadrature control I gain	6 bits	1 bits
Rate control P gain	400,000 bits	400,000 bits
Rate control I gain	300 bits	300 bits

**Table 10:** The measures of time constant and quality factor in according to target angle.

Target Azimuth Angle θ	Time Constant τ	Target Value
–90°	387 s	8.6635 × 10^6^
−60°	424 s	9.4987 × 10^6^
−30°	324 s	7.2620 × 10^6^
0°	281 s	6.2853 × 10^6^
30°	328 s	7.3470 × 10^6^
60°	374 s	8.3804 × 10^6^
90°	335 s	7.4885 × 10^6^

## Appendix A

An ideal Coriolis Vibrating Gyroscope (CVG) is a two-dimensional isotropic mass-spring system vibrating with the natural frequencies, $\omega_{1}$, $\omega_{2}$. In the presence of the angular velocity of system about the vertical axis of x′-axis and y′-axis (=Ω), the equations of motion of an ideal CVG are as follows [8]:

(A1) $$\ddot{x}\prime -2k\Omega \dot{y^{\prime }}-k\dot{\Omega }y^{\prime }+\left(\omega_{2}^{2}-k^{2}\Omega^{2}\right)x^{\prime }=f_{x\prime }$$

(A2) $$\ddot{y\prime }+2k\Omega \dot{x^{\prime }}+k\dot{\Omega }x^{\prime }+\left(\omega_{1}^{2}-k^{2}\Omega^{2}\right)y^{\prime }=f_{y\prime }$$

where k is the Brian coefficient(~0.3), $f_{x\prime }$ $f_{y\prime }$ is the linear acceleration of the x′-axis and y′-axis, $\Omega^{2}$, $\dot{\Omega }$ are the centrifugal and angular acceleration terms. In the presence of the angle by the unbalance between the x-axis and the major axis of resonance mode (=$\theta_{\omega })$, assuming that the x-axis and y-axis are assumed the axes where the excitation and sensing electrodes are aligned, the relations between x′, y′ axes and x, y axes are:

(A3) $$\left[\begin{array}{c} x\prime \\ y\prime \end{array}\right]=\left[\begin{array}{cc} cos\theta_{\omega } & sin\theta_{\omega }\\ -sin\theta_{\omega } & cos\theta_{\omega } \end{array}\right]\left[\begin{array}{c} x\\ y \end{array}\right]$$

Equations (A1) and (A2) can be arranged as follows:

(A4) $$\ddot{x}-2k\Omega \dot{y}-k\dot{\Omega }y+\left(\frac{\omega_{1}^{2}+\omega_{2}^{2}}{2}-k^{2}\Omega^{2}\right)x-\frac{\omega_{1}^{2}-\omega_{2}^{2}}{2}\left(xcos2\theta_{\omega }+ysin2\theta_{\omega }\right)=f_{x}$$

(A5) $$\ddot{y}+2k\Omega \dot{x}+k\dot{\Omega }x+\left(\frac{\omega_{1}^{2}+\omega_{2}^{2}}{2}-k^{2}\Omega^{2}\right)y+\frac{\omega_{1}^{2}-\omega_{2}^{2}}{2}\left(-xsin2\theta_{\omega }+ycos2\theta_{\omega }\right)=f_{y}$$

In the presence of the mismatch angle between the x-axis and the major axis of the linear damping(=$\theta_{\tau }$), the Equations (A4) and (A5) are as follows by coordinate transformation:

(A6) $$\ddot{x}-2k\Omega \dot{y}-k\dot{\Omega }y+\;\left(\frac{1}{\tau_{1}}+\frac{1}{\tau_{2}}\right)\dot{x}+\left(\frac{1}{\tau_{1}}-\frac{1}{\tau_{2}}\right)\left(\dot{x}cos2\theta_{\tau }+\dot{y}sin2\theta_{\tau }\right)+\left(\frac{\omega_{1}^{2}+\omega_{2}^{2}}{2}-k^{2}\Omega^{2}\right)x-\frac{\omega_{1}^{2}-\omega_{2}^{2}}{2}\left(xcos2\theta_{\omega }+ysin2\theta_{\omega }\right)=f_{x}$$

(A7) $$\ddot{y}+2k\Omega \dot{x}+k\dot{\Omega }x+\;\left(\frac{1}{\tau_{1}}+\frac{1}{\tau_{2}}\right)\dot{y}-\left(\frac{1}{\tau_{1}}-\frac{1}{\tau_{2}}\right)\left(-\dot{x}sin2\theta_{\tau }+\dot{y}cos2\theta_{\tau }\right)+\left(\frac{\omega_{1}^{2}+\omega_{2}^{2}}{2}-k^{2}\Omega^{2}\right)y+\frac{\omega_{1}^{2}-\omega_{2}^{2}}{2}\left(-xsin2\theta_{\omega }+ycos2\theta_{\omega }\right)=f_{y}$$

where $\tau_{1}$, $\tau_{2}$ are energy dissipation time constants. Since the coefficients are defined as follows, Equations (A6) and (A7) can be arranged as Equation (A9):

(A8) $$\omega^{2}=\frac{\omega_{1}^{2}+\omega_{2}^{2}}{2},\frac{1}{\tau }=\frac{1}{2}\left(\frac{1}{\tau_{1}}+\frac{1}{\tau_{2}}\right),\omega \Delta \omega =\frac{\omega_{1}^{2}-\omega_{2}^{2}}{2},\Delta \left(\frac{1}{\tau }\right)=\frac{1}{\tau_{1}}-\frac{1}{\tau_{2}}$$

(A9) $$\left[\begin{array}{c} \ddot{x}\\ \ddot{y} \end{array}\right]+\left[\begin{array}{cc} C_{11} & C_{12}-2k\Omega \\ C_{21}+2k\Omega & C_{22} \end{array}\right]\left[\begin{array}{c} \dot{x}\\ \dot{y} \end{array}\right]+\left[\begin{array}{cc} K_{11} & K_{12}\\ K_{21} & K_{22} \end{array}\right]\left[\begin{array}{c} x\\ y \end{array}\right]=\left[\begin{array}{c} f_{x}\\ f_{y} \end{array}\right]$$

where:

$$C_{11}=\frac{2}{\tau }+\Delta \left(\frac{1}{\tau }\right)\cos2\theta_{\tau },C_{12}=C_{21}=\Delta \left(\frac{1}{\tau }\right)\sin2\theta_{\tau },C_{22}=\frac{2}{\tau }-\Delta \left(\frac{1}{\tau }\right)\cos2\theta_{\tau }$$

$$K_{11}=\omega^{2}-\omega \Delta \omega \cos2\theta_{\omega },K_{12}=K_{21}=-\omega \Delta \omega \sin2\theta_{\omega },K_{22}=\omega^{2}+\omega \Delta \omega \cos2\theta_{\omega }$$

## Appendix B

Assuming that $x_{ct}\left(t\right)=\int_{C_{1}T_{0}\left(n-1\right)}^{t}f\left(\tau \right)h\left(t-\tau \right)d\tau$, $x_{pt}\left(t\right)=\int_{C_{1}T_{0}\left(k-1\right)}^{C_{2}T_{0}+C_{1}T_{0}\left(k-1\right)}f\left(\tau \right)h\left(t-\tau \right)d\tau$, the calculation results are follows. As in this moment, the Q-factor is high, we can assume that $\omega_{d}≅\omega$:

(B1) $$x_{ct}\left(t\right)=\frac{f_{0}}{m_{n}\omega_{d}}e^{-\frac{\omega }{2Q}t}\int_{C_{1}T_{0}\left(n-1\right)}^{t}\cos\omega \tau \;e^{\frac{\omega }{2Q}\tau }\sin\omega_{d}\left(t-\tau \right)d\tau$$

(B2) $$x_{ct}\left(t\right)=\frac{f_{0}}{m_{n}\omega_{d}}e^{-\frac{\omega }{2Q}t}\left(\sin\omega_{d}t\int_{C_{1}T_{0}\left(n-1\right)}^{t}e^{\frac{\omega }{2Q}\tau }\cos\omega \tau \cos\omega_{d}\tau d\tau -\int_{C_{1}T_{0}\left(n-1\right)}^{t}e^{\frac{\omega }{2Q}\tau }\cos\omega \tau \sin\omega_{d}\tau d\tau \right)$$

(B3) $$\begin{array}{l} \int_{C_{1}T_{0}\left(n-1\right)}^{t}e^{\frac{\omega }{2Q}\tau }\cos\omega \tau \cos\omega_{d}\tau d\tau ={\left[\frac{e^{\frac{\omega }{2Q}\tau }\cos\omega \tau }{{\left(\frac{\omega }{2Q}\right)}^{2}+4\omega^{2}}\left(\frac{\omega }{2Q}\cos\omega \tau +2\omega \sin\omega t\right)+\frac{4\omega Qe^{\frac{\omega }{2Q}\tau }}{{\left(\frac{\omega }{2Q}\right)}^{2}+4\omega^{2}}\right]}_{C_{1}T_{0}}^{t}\\ \;=\;\frac{e^{\frac{\omega }{2Q}t}}{{\left(\frac{\omega }{2Q}\right)}^{2}+4\omega^{2}}\left(4\omega Q+\frac{\omega }{2Q}\cos^{2}\omega t+\omega \sin2\omega t\right)-\frac{\left(\frac{\omega }{2Q}+4\omega Q\right)e^{\frac{C_{1}\pi \left(n-1\right)}{Q}}}{{\left(\frac{\omega }{2Q}\right)}^{2}+4\omega^{2}} \end{array}$$

(B4) $$\int_{C_{1}T_{0}\left(n-1\right)}^{t}e^{\frac{\omega }{2Q}\tau }\cos\omega \tau \sin\omega_{d}\tau d\tau =\frac{1}{2}\int_{C_{1}T_{0}\left(n-1\right)}^{t}e^{\frac{\omega }{2Q}\tau }\sin2\omega \tau d\tau =\frac{1}{2}{\left[\frac{e^{\frac{\omega }{2Q}\tau }\left(\frac{\omega }{2Q}\sin2\omega \tau -2\omega \cos2\omega \tau \right)}{{\left(\frac{\omega }{2Q}\right)}^{2}+4\omega^{2}}\right]}_{C_{1}T_{0}}^{t}\;=\frac{1}{{\left(\frac{\omega }{2Q}\right)}^{2}+4\omega^{2}}\left[e^{\frac{\omega }{2Q}t}\left(\frac{\omega }{4Q}\sin2\omega t-\omega \cos2\omega t\right)+\omega e^{\frac{C_{1}\pi \left(n-1\right)}{Q}}\right]$$

Equations (B3) and (B4) are inserted to Equation (B2), it can be arranged as follows:

(B5) $$x_{ct}\left(t\right)=\frac{f_{0}/m_{n}\omega_{d}}{{\left(\frac{\omega }{2Q}\right)}^{2}+4\omega^{2}}\left[\left(4\omega Q\sin\omega t+\omega \cos\omega t\right)\left(1-e^{\frac{2C_{1}\pi \left(n-1\right)-\omega t}{2Q}}\right)-\frac{\omega }{2Q}\sin\omega t\;e^{\frac{2C_{1}\pi \left(n-1\right)-\omega t}{2Q}}\right]$$

(B6) $$x_{pt}\left(t\right)=\frac{f_{0}}{m_{n}\omega_{d}}e^{-\frac{\omega }{2Q}t}\left(\sin\omega_{d}t\int_{C_{1}T_{0}\left(k-1\right)}^{C_{2}T_{0}+C_{1}T_{0}\left(k-1\right)}e^{\frac{\omega }{2Q}\tau }\cos\omega \tau \cos\omega_{d}\tau d\tau -\cos\omega_{d}t\int_{C_{1}T_{0}\left(k-1\right)}^{C_{2}T_{0}+C_{1}T_{0}\left(k-1\right)}e^{\frac{\omega }{2Q}\tau }\cos\omega \tau \sin\omega_{d}\tau d\tau \right)$$

(B7) $$\begin{array}{l} \int_{C_{1}T_{0}\left(k-1\right)}^{C_{2}T_{0}+C_{1}T_{0}\left(k-1\right)}e^{\frac{\omega }{2Q}\tau }\cos\omega \tau \cos\omega_{d}\tau d\tau =\;\frac{e^{\frac{\pi \left[C_{2}+C_{1}\left(k-1\right)\right]}{Q}}}{{\left(\frac{\omega }{2Q}\right)}^{2}+4\omega^{2}}\left(4\omega Q+\frac{\omega }{2Q}\right)-\frac{\left(\frac{\omega }{2Q}+4\omega Q\right)e^{\frac{C_{1}\pi \left(k-1\right)}{Q}}}{{\left(\frac{\omega }{2Q}\right)}^{2}+4\omega^{2}}\\ \;=\;\frac{\left(\frac{\omega }{2Q}+4\omega Q\right)e^{\frac{C_{1}\pi \left(k-1\right)}{Q}}}{{\left(\frac{\omega }{2Q}\right)}^{2}+4\omega^{2}}\left(e^{\frac{C_{2}\pi }{Q}}-1\right) \end{array}$$

(B8) $$\int_{C_{1}T_{0}\left(k-1\right)}^{C_{2}T_{0}+C_{1}T_{0}\left(k-1\right)}e^{\frac{\omega }{2Q}\tau }\cos\omega \tau \cos\omega_{d}\tau d\tau =\frac{1}{{\left(\frac{\omega }{2Q}\right)}^{2}+4\omega^{2}}[-\omega e^{\frac{\pi \left[C_{2}+C_{1}\left(k-1\right)\right]}{Q}}+\omega e^{\frac{\pi C_{1}\left(k-1\right)}{Q}}]=\frac{\omega e^{\frac{C_{1}\left(k-1\right)}{Q}}}{{\left(\frac{\omega }{2Q}\right)}^{2}+4\omega^{2}}\left(1-e^{\frac{\pi C_{2}}{Q}}\right)$$

Equations (B7) and (B8) are inserted to Equation (B6), it can be arranged as follows:

(B9) $$x_{pt}\left(t\right)=\frac{f_{0}/m_{n}\omega_{d}}{{\left(\frac{\omega }{2Q}\right)}^{2}+4\omega^{2}}e^{\frac{\pi C_{1}\left(k-1\right)}{Q}}\left(e^{\frac{2\pi C_{2}-\omega t}{2Q}}-e^{-\frac{\omega t}{2Q}}\right)\left[\left(\frac{\omega }{2Q}+4\omega Q\right)\sin\omega_{d}t+\omega \cos\omega_{d}t\right]$$

## Appendix C

The time response of SDOF spring-damper system by the switched excitation can be arranged as follows:

Case a. $C_{1}T_{0}\left(n-1\right)<t\leq C_{2}T_{0}+C_{1}T_{0}\left(n-1\right),n=1,2,\cdots ,\infty$
  (C1) $$x\left(t\right)=\frac{\frac{f_{0}}{m_{n}\omega_{d}}}{{\left(\frac{\omega }{2Q}\right)}^{2}+4\omega^{2}}\{\overset{k=n-1}{\underset{k=1}{\sum }}e^{\frac{\pi C_{1}\left(k-1\right)}{Q}}\left(e^{\frac{2\pi C_{2}-\omega t}{2Q}}-e^{-\frac{\omega t}{2Q}}\right)\left[\left(\frac{\omega }{2Q}+4\omega Q\right)\sin\omega_{d}t+\omega \cos\omega_{d}t\right]\;+\left[\left(4\omega Q\sin\omega t+\omega \cos\omega t\right)\left(1-e^{\frac{2\pi C_{1}\left(n-1\right)-\omega t}{2Q}}\right)-\frac{\omega }{2Q}\sin\omega t\;e^{\frac{2\pi C_{1}\left(n-1\right)-\omega t}{2Q}}\right]\}$$
Case b. $C_{2}T_{0}\left(n-1\right)+C_{1}T_{0}<t\leq 2C_{1}T_{0}n,n=1,2,\cdots ,\infty$
  (C2) $$x\left(t\right)=\frac{\frac{f_{0}}{m_{n}\omega_{d}}}{{\left(\frac{\omega }{2Q}\right)}^{2}+4\omega^{2}}\overset{k=n}{\underset{k=1}{\sum }}e^{\frac{\pi C_{1}\left(k-1\right)}{Q}}\left(e^{\frac{2\pi C_{2}-\omega t}{2Q}}-e^{-\frac{\omega t}{2Q}}\right)\left[\left(\frac{\omega }{2Q}+4\omega Q\right)\sin\omega_{d}t+\omega \cos\omega_{d}t\right]$$

Assumed that $C_{x}=\frac{\frac{f_{0}}{m_{n}\omega_{d}}}{{\left(\frac{\omega }{2Q}\right)}^{2}+4\omega^{2}},1+\frac{1}{8Q^{2}}≅1$, $\sqrt{{\left(\frac{1}{2Q}+4Q\right)}^{2}+1}≅\sqrt{16Q^{2}+1}$(Q≫1), The equation related A(t) is arranged as follows:

Case a. $C_{1}T_{0}\left(n-1\right)<t\leq C_{2}T_{0}+C_{1}T_{0}\left(n-1\right),n=1,2,\cdots ,\infty$
  (C3) $$A\left(t\right)=C_{x}\omega \sqrt{16Q^{2}+1}\left[\overset{k=n-1}{\underset{k=1}{\sum }}\left(e^{\frac{\pi C_{2}}{Q}}-1\right)e^{\frac{2\pi C_{1}\left(k-1\right)-\omega t}{2Q}}+\left(1-e^{\frac{2\pi C_{1}\left(n-1\right)-\omega t}{2Q}}\right)\right]$$
Case b. $C_{2}T_{0}\left(n-1\right)+C_{1}T_{0}<t\leq 2C_{1}T_{0}n,n=1,2,\cdots ,\infty$
  (C4) $$A\left(t\right)=C_{x}\omega \sqrt{16Q^{2}+1}\overset{k=n}{\underset{k=1}{\sum }}\left(e^{\frac{\pi C_{2}}{Q}}-1\right)e^{\frac{2\pi C_{1}\left(k-1\right)-\omega t}{2Q}}$$

Based on the above equations, the mean amplitude $\bar{A}\left(t\right)$ while $0<t\leq C_{1}T_{0}$ is calculated as follows:

(C5) $$\bar{A}\left(t\leq C_{1}T_{0}\right)=C_{x}\omega \sqrt{16Q^{2}+1}\frac{Q}{\pi C_{1}}\left(e^{\frac{\pi C_{2}}{Q}}-1\right)\left[1-e^{-\frac{\omega t}{2Q}}\left(1+\frac{C_{1}-C_{2}}{2C_{1}Q}t\right)\right]$$

The amplitude while $t\leq C_{1}T_{0}+C_{2}T_{0}$ is as follows:

(C6) $$A\left(t\leq C_{1}T_{0}+C_{2}T_{0}\right)=C_{x}\omega \sqrt{16Q^{2}+1}\left\{\frac{Q}{\pi C_{1}}\left(e^{\frac{\pi C_{2}}{Q}}-1\right)\left[1-e^{-\frac{\omega t}{2Q}}\left(1+\frac{C_{1}-C_{2}}{2C_{1}Q}t\right)\right]+\left(1-e^{\frac{2\pi C_{1}-\omega t}{2Q}}\right)\right\}$$

The amplitude while $t\leq 2C_{1}T_{0}$ is as follows:

(C7) $$A\left(t\leq 2C_{2}T_{0}\right)=C_{x}\omega \sqrt{16Q^{2}+1}\left\{\frac{Q}{\pi C_{1}}\left(e^{\frac{\pi C_{2}}{Q}}-1\right)\left[1-e^{-\frac{\omega t}{2Q}}\left(1+\frac{C_{1}-C_{2}}{2C_{1}Q}t\right)\right]+\left(e^{\frac{\pi C_{2}}{Q}}-1\right)e^{\frac{2\pi C_{1}-\omega t}{2Q}}\right\}$$

Based on the above equations, the mean amplitude $\bar{A}\left(t\right)$ while $0<t\leq 2C_{1}T_{0}$ is calculated as follows:

(C8) $$\bar{A}\left(t\leq 2C_{1}T_{0}\right)=C_{x}\omega \sqrt{16Q^{2}+1}\frac{Q}{\pi C_{1}}\left(e^{\frac{\pi C_{2}}{Q}}-1\right)\left\{1-e^{-\frac{\omega t}{2Q}}\left[1+\frac{C_{1}-C_{2}}{2C_{1}Q}t+\frac{1}{2}{\left(\frac{C_{1}-C_{2}}{2C_{1}Q}t\right)}^{2}\right]\right\}$$

With the same method, the mean amplitude by operation cycle is calculated as follows:

(C9) $$\bar{A}\left(t\leq 3C_{1}T_{0}\right)=C_{x}\omega \sqrt{16Q^{2}+1}\frac{Q}{\pi C_{1}}\left(e^{\frac{\pi C_{2}}{Q}}-1\right)\left\{1-e^{-\frac{\omega t}{2Q}}\left[1+\frac{C_{1}-C_{2}}{2C_{1}Q}t+\frac{1}{2}{\left(\frac{C_{1}-C_{2}}{2C_{1}Q}t\right)}^{2}+\frac{1}{6}{\left(\frac{C_{1}-C_{2}}{2C_{1}Q}t\right)}^{3}\right]\right\}$$

(C10) $$\bar{A}\left(t\leq nC_{1}T_{0}\right)=C_{x}\omega \sqrt{16Q^{2}+1}\frac{Q}{\pi C_{1}}\left(e^{\frac{\pi C_{2}}{Q}}-1\right)\left\{1-e^{-\frac{\omega t}{2Q}}\left[1+\frac{C_{1}-C_{2}}{2C_{1}Q}t+\frac{1}{2}{\left(\frac{C_{1}-C_{2}}{2C_{1}Q}t\right)}^{2}+\cdots +\frac{1}{n!}{\left(\frac{C_{1}-C_{2}}{2C_{1}Q}t\right)}^{n}\right]\right\}$$

The Taylor series included in above equation can be expressed briefly as follows:

(C11) $$1+\frac{C_{1}-C_{2}}{2C_{1}Q}t+\frac{1}{2}{\left(\frac{C_{1}-C_{2}}{2C_{1}Q}t\right)}^{2}+\cdots +\frac{1}{n!}{\left(\frac{C_{1}-C_{2}}{2C_{1}Q}t\right)}^{n}=e^{\frac{\left(C_{1}-C_{2}\right)/C_{1}}{2Q}t}$$

Therefore, the time response characteristics of the mean amplitude by the switched excitation are as follows:

(C12) $$\bar{A}\left(t\right)=\frac{Q}{\pi C_{1}}\left(e^{\frac{\pi C_{2}}{Q}}-1\right)\frac{f_{0}Q}{m_{n}\omega^{2}}\left[1-e^{-\frac{\left(\omega -\frac{C_{1}-C_{2}}{C_{1}}\right)}{2Q}t}\right]$$
